# Robotic-Assisted Bladder Neck Procedures for Incontinence in Pediatric Patients

**DOI:** 10.3389/fped.2019.00172

**Published:** 2019-05-07

**Authors:** Patricio C. Gargollo, Lindsay A. White

**Affiliations:** ^1^Division of Pediatric Urology, The Mayo Clinic and Mayo Medical School, Rochester, MN, United States; ^2^The University of Washington School of Medicine, Seattle, WA, United States

**Keywords:** robotic surgery, urinary incontinence, bladder neck reconstruction, appendicovesicostomy, artificial urinary sphincter, bladder neck sling, bladder neck closure

## Abstract

**Purpose:** To review the current status of bladder neck procedures for incontinence in pediatric patients, focusing on the increasing role of robotic-assisted laparoscopic surgical techniques.

**Methods:** A comprehensive review of the literature on open and robotic-assisted bladder neck procedures was conducted, with a focus on articles published in the last 20 years. This data was subsequently compared with published results from robotic-assisted bladder neck reconstruction series completed at our institution.

**Results:** The principal bladder neck procedures for incontinence in pediatric patients include: Artificial Urinary Sphincter, Bladder Neck Sling, Bladder Neck Closure and Bladder Neck Reconstruction. Continence rates range from 60 to 100% with a lack of expert consensus on the preferred procedure (or combination of procedures). Robotic-assisted approaches are associated with longer operative times, especially early in the surgical experience, but demonstrate equivalent continence rates with potential benefits including: low intraoperative blood loss, improved cosmesis, and decreased intra-abdominal adhesion formation.

**Conclusions:** Robotic-assisted procedures of the bladder neck are safe, feasible, follow the same steps and principles as those of open surgery and produce equivalent continence rates. Robotic-assisted techniques can be adapted to a variety of bladder neck procedures and safely expanded to selected patients with previous open abdominal surgery.

## Introduction

Minimally invasive techniques are at the forefront of urologic surgery. As advances in laparoscopic instrumentation and robotic technology continue, the use of these techniques in the pediatric population has expanded. Robotic-assisted procedures in the pediatric population are well established for nephrectomy, pyeloplasty, ureteral reimplantation, and bladder surgery, but the use of this technology remains limited in more complex reconstructive cases. Longer operative times are often cited as a barrier to the use of minimally invasive techniques in complex reconstruction, but it has been demonstrated that much of the difference in operative time can be mitigated with increased surgeon experience (1, 2). Even with longer operative times, advantages over open surgery may include lower intraoperative blood loss, decreased post-operative narcotic use, and superior cosmesis (3, 4). These benefits have the potential to be accentuated in the pediatric population due to the relatively confined working spaces and a heightened awareness of cosmesis. In addition, the potential for decreased intra-abdominal adhesion formation in laparoscopic and robotic procedures, as compared to open, cannot be discounted. In patient populations that are at high risk of undergoing multiple lifetime abdominal surgeries, the benefits of decreased adhesions could have significant implications in reducing both technical difficulty and operative time in re-operation (5, 6).

Furthermore, as the use of minimally invasive surgical techniques increases, so too does the pool of patients considered to be candidates for this approach. While previous open abdominal surgery (PAS) was traditionally considered a relative contraindication to a laparoscopic or robotic-assisted approach, emerging studies indicate safety and efficacy of complex robotic reconstructions in these populations (1). In this review, we will discuss the surgical interventions available for pediatric incontinence, the increasing role of robotic-assisted techniques, complex reconstruction of the bladder neck, and our cumulative experience and approach to treating these patients.

## Surgical Intervention for Urinary Incontinence

Urine leakage in the absence of a detrusor contraction, regardless of the primary cause (exstrophy/epispadias, cloacal anomalies, or neurogenic bladder secondary to spinal cord injury or dysraphisms) is the definition of an incompetent urinary sphincter mechanism (7). The basic goal behind surgical procedures to address this incompetent sphincter mechanism is to tighten the bladder outlet. This can be achieved in many ways including: placement of an artificial urinary sphincter (AUS), placement of a bladder neck sling (BNS), bladder neck reconstruction (BNR), and bladder neck closure (BNC). We will review the current status of each of these surgical interventions for incontinence, and more specifically, how these procedures are being adapted for robotic-assisted laparoscopic surgery (RALS). Whether or not a concomitant bladder augmentation procedure should be performed with the procedures listed above is a highly contested topic and beyond the scope of this review, and thus will not be covered here.

## Artificial Urinary Sphincter

The AUS has been used for surgical intervention in pediatric incontinence for decades. The historical benefits of AUS placement include the ability to maintain the potential for spontaneous voiding, decreasing the percentage of patients required to complete obligatory clean intermittent catheterization (CIC), and the potential to delay, or avoid, fixed resistance procedures such as BNS, BNR, or BNC. Continence rates following AUS placement in the pediatric population have remained similar across decades with Simeoni et al. reporting overall success rates of 77% in 1996 (8) and Catti et al. demonstrating similar results with 73% of patients achieving continence in 2008 (9). Despite good continence, both studies also demonstrated relatively high complication rates, with device removal required in 19 and 20% of patients, respectively (8, 9). In addition, post-operative changes in detrusor dynamics following AUS placement are concerning as they have been shown to lead to renal failure in 16% of patients and the need for augmentation cystoplasty in 31% of patients (10).

In patients with previous surgery of the bladder neck or proximal urethra success rates of AUS placement become extremely variable. Castera et al. evaluated AUS placement in 49 patients with 67% of all patients achieving continence, but further comparison demonstrated that of patients with no previous surgery (*n* = 25), 86% achieved urinary continence, whereas only 37.5% of patients who had undergone prior bladder neck surgical procedures (*n* = 8) achieved continence (11). Ruiz et al. followed this study with examination of AUS placement in 23 patients without spina bifida, but with previous surgery of the bladder neck or proximal urethra and achieved continence in 87% of the 19 sphincters that remained in place (86.3% survival rate) (12). Levesque et al. looked beyond efficacy to evaluate long-term survival of the AUS in the pediatric population and found that the mean survival time of the prostheses was 12.5 years (95% CI, 10.3–14.6), and was independent of patient sex or incontinence etiology (13). In addition, Bauer conducted a meta-analysis encompassing 585 pediatric patients from reported series of AUS placement between 1996 and 2006 and found that 32% of patients voided spontaneously to completion without the aid of CIC, demonstrating that AUS may provide large advantages over other bladder outlet procedures for patients in which spontaneous voiding is a priority (10).

Recently, Moscardi et al. reported the first described case of robotic-assisted laparoscopic (RAL)-AUS placement in the pediatric population. The procedure was safely accomplished, and the patient remained continent between volitional voids at 3-month follow-up (14). This report demonstrates that RAL-AUS placement is feasible in the pediatric population, which may be advantageous in facilitating concomitant intra-abdominal procedures, but further experience will be required to compare the safety, efficacy, and long-term outcomes, as it remains unclear if robotic-assistance will affect previously reported continence or complication rates.

## Bladder Neck Sling

Comparison of published data regarding the effectiveness of BNS for pediatric incontinence is challenging because most published series include patients with previous or concurrent procedures. In a review article from 2000, Kryger et al. reported overall continence for fascial sling placement varying from 40 to 100%, but with very limited follow-up (15). Snodgrass and colleagues completed BNS placement with appendicovesicostomy (APV) in 30 children with satisfactory continence (defined as 2 or fewer damp pads daily) achieved in 83% of patients (16). In addition, Snodgrass and Barber compared success rates of 34 consecutive patients with neurogenic incontinence receiving BNS alone and 37 patients receiving BNS plus Leadbetter-Mitchell (LM) BNR. They found that 46% of BNS alone cases did not require pads post-operatively whereas 82% of BNS plus LM BNR did not require pads (17). In addition, 6-month follow-up demonstrated that no patients had hydronephrosis and only 2 patients (BNS plus LM BNR) had transient low-grade reflux (grade I or II) on postoperative urodynamics, which resolved on subsequent studies 2 and 4 months later (17). It was initially believed that this may represent BNS superiority for preservation of the upper tracts, but this idea was later called into question by Grimsby et al. when they found that upon long-term assessment, 30% of these patients had postoperative vesicoureteral reflux (VUR)/hydronephrosis and 17% required ureteral reimplantation or subureteral injection of bulking agent (18). This further demonstrates the effects of limited clinical series and limited follow-up when comparing the success and long-term safety of BNS placement to alternative bladder outlet procedures.

These same confounders and limitations exist when comparing open vs. robotic-assisted BNS placement. Rare reports of BNS placement without concurrent BNR or APV exist, again making it difficult to compare to the open series described above. This author has extensive experience with RAL-BNS placement, but as this procedure is performed concurrently with BNR and APV, results will be discussed in subsequent sections. Finally, due to the superior continence rates now being achieved with BNS placement plus concurrent procedures, it is unlikely that the success of BNS alone will be able to be evaluated without these confounding factors.

## Bladder Neck Closure

Closure of the bladder neck is perhaps the most radical option for achieving continence, as it is irreversible and requires compliance with obligatory CIC of a cutaneous stoma. Bergman et al. showed an 88% dry rate in a retrospective review of patients with mixed etiology incontinence who failed medical therapy and underwent bladder neck closure as their primary surgery (19). This is similar to findings by Liard et al. who evaluated 21 patients with BNC as primary surgical therapy and showed an 80% dry rate (20). Another retrospective study by Hoebeke et al. evaluated 17 children undergoing BNC and demonstrated a dry rate of 100% but difficulty with catheterization in 47% of patients (21). Kavanagh et al. more recently reviewed 28 consecutive patients undergoing BNC and found an initial success rate in 96.4% (27 of 28) of patients. Importantly, 68% of these patients had undergone previous unsuccessful bladder neck procedures, demonstrating safety and efficacy of BNC as a secondary surgical intervention, though a relatively high total surgical re-intervention rate of 39.3% (11 of 28) should be noted (22).

Robotic BNC has been reported in the literature (23), but all current reports include BNC as a concomitant procedure performed as part of a larger study. Murthy et al. preformed 4 RAL-BNC in conjunction with RAL-augmentation ileocystoplasty and APV with good short-term results, but updated interim results revealed that 3 of 4 RAL-BNC required further surgical intervention due to dehiscence (23). The feasibility of RAL-BNC is expected given the excellent exposure to the pelvis and bladder provided by a robotic-assisted approach, but to date published literature on RALS-BNC in the pediatric population is too limited to confirm safety, provide continence rates or compare to open series.

## Bladder Neck Reconstruction

Various reconstructive procedures and operative techniques are available to increase the resistance at the bladder outlet, including the Young-Dees Leadbetter (YDL), the Pippi-Salle, the Kropp repair, and the modified LM repair. To date, no single technique has demonstrated consistently superior outcomes, in large part due to multiple limitations of the published literature including: retrospective studies with significant confounders (various primary diagnoses, previous or concurrent procedures, use of augmentation cystoplasty), non-standardized protocols, and multiple definitions of what constitutes urinary continence. Cole et al. compared these techniques in a retrospective review of 49 continence procedures for patients with varying etiologies of incontinence and found continence rates of 79% for YDL and 75% for Kropp and Pippi-Salle repairs (24). Another retrospective review of 18 children with neurogenic incontinence showed a dry rate (4h or more between catheterizations) of 61% following Pippi-Salle reconstruction (25). As previously described, Snodgrass and Barber were able to achieve 82% continence rate with LM BNR plus BNS with a mean follow-up of 13 months, with 60% still dry at maximum follow-up of 55 months (17). Despite the limitations in evaluation, these open BNR series demonstrate continence rates ranging from 50 to 85% (2).

Grimsby and colleagues reviewed the perioperative and short-term outcomes between 26 open and 19 RAL-BNRs. They found that while operative time was significantly longer in the robotic group (8.2 vs. 4.5h, *p* < 0.001), there was no difference in length of stay (median 4 days), acute complications or continence outcomes (26). Re-operation rates were slightly less in the robotic group with 56% (14 patients) in the open group undergoing a total of 23 subsequent surgeries, compared to 42% (8 patients) in the robotic group undergoing 12 additional procedures (*p* = 0.5), although follow-up was longer in the open group (26). Operative time at re-operation was not reported in this study, but a previous report by the same authors comparing 28 open APVs and 39 RAL-APVs, found that while there was no difference in number of complications (*p* = 0.788) or number of re-operations (*p* = 0.791), the first re-operation had a significantly shorter operative time in the robotic group (6).

**Figure 1:**
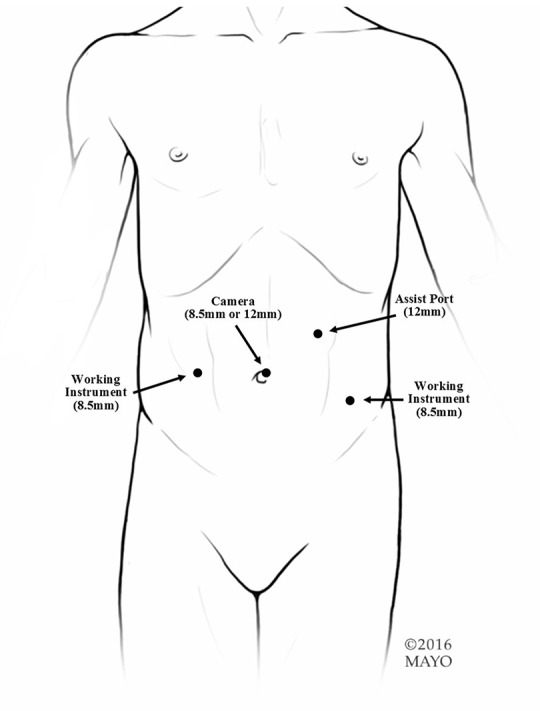
Port placement. We use an 8.5-mm or 12-mm camera and two 8.5-mm working ports. If any bowel work is going to be performed or if a sling is going to be used, we suggest a 12-mm assist in the left upper quadrant. If just an APV is going to be performed, a 5-mm assist port can be used.

**Figure 2:**
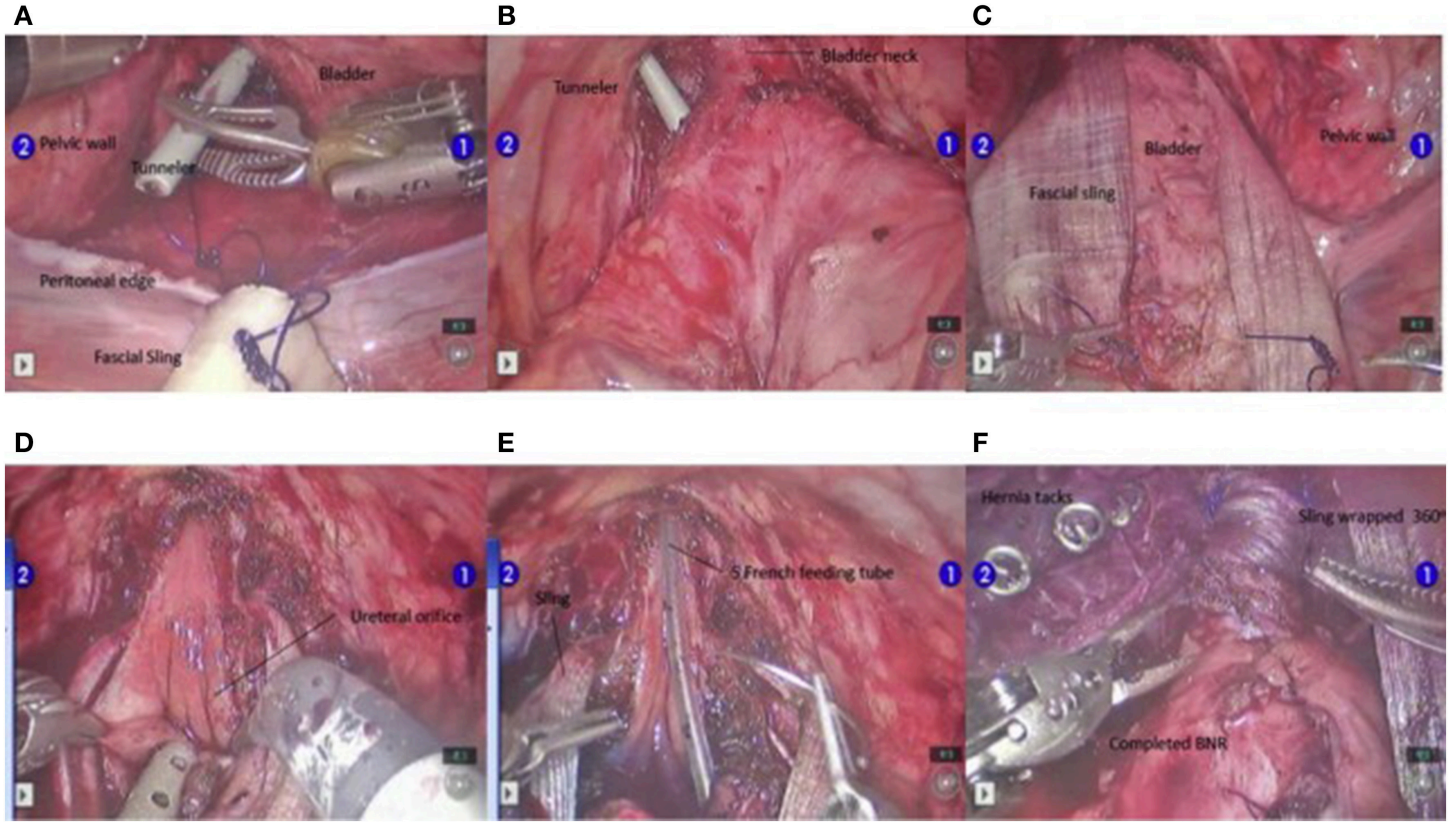
Steps in the BNR. The tunnelers are passed ventrally from the posterior bladder dissection into the developed space of Retzius **(A,B)**. Once the sling is passed from posterior to anterior **(C)**, the urethra is unroofed up through the bladder neck to the level of the interureteric ridge **(D)**. At this point, the Foley catheter is exchanged for a 5-F feeding tube, and the urethra is retubularized in 2 layers with a running simple suture of 4-0 Vicryl followed by 3-0 Vicryl **(E)**. After the LM repair is completed, the sling is tightly wrapped 360° and attached to the pubic bone using 6 screws from a hernia tacker **(F)**. Permission for use of this figure has been obtained from Elsevier Publishing Company. The originally published form can be found at Gargollo (2).

At our institution, the procedure of choice for management of neurogenic bladder with persistent urinary incontinence (despite CIC and anticholinergic therapy) includes creation of a RAL-APV (or Monti channel when the appendix is inadequate) and LM BNR along with BNS placement. This combination of procedures and the specific technique to complete them robotically was first described by this author in “Robotic-assisted bladder neck repair: feasibility and outcomes” in 2015, with relatively few adjustments made to the procedure since this date (2). Our RAL approach to BNR allows for minimal incisions, limited to an inverted “V”-shaped incision in the umbilicus, an assist port in the left upper quadrant and two 8.5-mm working ports, as represented in Figure 1. In addition, this technique facilitates dissection, creating excellent exposure to the pelvis and the bladder, and providing the ability to accommodate any type of bladder neck repair (e.g., LM, Pippi-Salle, Kropp and YDL). As previously described, our institution utilizes retrovesicular placement of the sling, LM BNR, retubularization of the urethra around a 5-Fr feeding tube and 360 degree sling wrapping, as shown in Figure 2. We then hitch the bladder to the anterior abdominal wall prior to creation of the APV.

Using this technique in 38 patients, 82% (*n* = 31) became completely dry during the day on CIC every 3h and of the 7 patients who were wet, 4 were non-compliant with CIC (2). This is consistent with the 85.2% initial continence rate reported by Gundeti et al. in a multi-institutional review of 88 patients undergoing RAL-APV and various concomitant bladder neck procedures (including BNR in 21, BNS in 17, and BNC in 4) (27).

Our mean operative time for this reconstruction is 5.8h (3.6–12.25h), with longer operative times being significantly higher in the first 10 vs. the last 28 cases (*P* = 0.0001), demonstrating the ability to mitigate some of the increase in operative time with increased surgeon experience (2). In follow-up, 5.3% (*n* = 2) required augmentation cystoplasty due to the post-operative development of decreased bladder compliance, 10.5% (*n* = 4) developed *de novo* reflux (grades 2 and 3), and 5.3% (*n* = 2) developed bladder stones (2). These complications are consistent with those seen in the bladder outlet procedures described previously, but to date, have occurred at a relatively lower rate, though direct comparison is challenged by multiple confounders.

Finally, the majority of the BNR procedures described above have occurred as primary surgical intervention in patients with neurogenic bladder, but the techniques utilized in this repair have already been expanded to include a broader patient population. Last year, our institution presented the first RAL-BNR in a patient with classic bladder exstrophy at the American Urologic Association Annual Meeting (Gargollo et al., under review). This year, our institution published the first study demonstrating the feasibility and safety of RAL-BNR in patients with PAS. In 36 patients with PAS, the mean operative time was 8.2h, with the first 18 cases taking significantly longer than the last 18 cases (mean 9.1h vs. mean 7.5h, *p* = 0.002), again demonstrating a learning curve for this procedure. Throughout this study, there were 5 cases that were converted to open due to failure to progress. All conversions were secondary to intra-abdominal adhesions and all occurred in patients with multiple previous ventriculoperitoneal (VP) shunt revisions. At a mean follow-up of 2 years (range 2–36 months), 5.5% (*n* = 2) of patients who had previously undergone a BNR required a BNC for persistent incontinence and 8% (*n* = 3) underwent surgery for bladder calculi. While further studies comparing open and robotic approaches to BNR are needed in patients with PAS to determine equivalency or superiority of one approach over the other, our initial reports to date of RAL-BNR appear promising.

## Conclusion

RAL surgical techniques have immense potential in the surgical treatment of pediatric incontinence. Our review demonstrates that RAL techniques can be adapted to a variety of procedure types with equivalent continence rates. In addition, the added benefits of improved cosmesis, less intraoperative blood loss, less post-operative pain and decreased adhesion formation make a robotic approach the preference at our institution. Longer operative times can be expected, especially early in the surgical experience, but over time, operative times become significantly shorter and more similar to the duration expected for traditional open surgery for these procedures. Our data also support the feasibility and safety of expanding the range of RAL surgical candidates to include selected patients with PAS. Overall, while there is still a lack of expert consensus on the preferred reconstructive procedure (or combination of procedures) for the treatment of pediatric incontinence, it appears that RAL surgical techniques will continue to push the frontier.

## Author Contributions

PG conceived of the presented idea, developed the surgical technique described, participated in drafting of the manuscript and critically revised the manuscript for important intellectual content. LW completed the comprehensive literature review, participated in drafting of the manuscript and formatted the manuscript for submission, under supervision of PG. All authors reviewed and approved the final manuscript.

### Conflict of Interest Statement

The authors declare that the research was conducted in the absence of any commercial or financial relationships that could be construed as a potential conflict of interest.

## Abbreviations

APV: Appendicovesicostomy
AUS: Artificial Urinary Sphincter
BNC: Bladder Neck Closure
BNR: Bladder Neck Reconstruction
BNS: Bladder Neck Sling
CIC: Clean Intermittent Catheterization
LM: Leadbetter/Mitchell
PAS: Previous open abdominal surgery
RAL-AUS: Robotic-assisted laparoscopic artificial urinary sphincter
RALS: Robotic-assisted laparoscopic surgery.
